# GV‐971 attenuates the progression of neuromyelitis optica in murine models and reverses alterations in gut microbiota and associated peripheral abnormalities

**DOI:** 10.1111/cns.14847

**Published:** 2024-07-07

**Authors:** Xinying Yang, Zhongheng Zhangyi, Aisong Yu, Qinming Zhou, Aihua Xia, Ji Qiu, Meixiang Cai, Xingkun Chu, Liang Li, Zhengnan Feng, Zhiyu Luo, Guangqiang Sun, Jing Zhang, Meiyu Geng, Sheng Chen, Zuoquan Xie

**Affiliations:** ^1^ State Key Laboratory of Drug Research Shanghai Institute of Materia Medica, Chinese Academy of Sciences Shanghai China; ^2^ Shanghai Green Valley Pharmaceutical Co., Ltd Shanghai China; ^3^ Department of Neurology, Ruijin Hospital Shanghai Jiaotong University School of Medicine Shanghai China; ^4^ Shandong Laboratory of Yantai Drug Discovery Bohai Rim Advanced Research Institute for Drug Discovery Yantai Shandong China; ^5^ Co‐innovation Center of Neuroregeneration Nantong University Nantong China

**Keywords:** gut microbiota, GV‐971, neuroinflammation, Neuromyelitis optica spectrum disorders, NMO‐IgG

## Abstract

**Aims:**

Growing evidence suggests that an imbalanced gut microbiota composition plays a crucial role in the development of neuromyelitis optica spectrum disorders (NMOSD), an inflammatory demyelinating disease primarily affecting the optic nerves and central nervous system (CNS). In light of this, we explored the potential therapeutic benefits of GV‐971 in NMOSD. GV‐971 is a drug used for treating mild‐to‐moderate Alzheimer's disease, which targets the gut‐brain axis and reduces neuroinflammation.

**Methods:**

To evaluate GV‐971's effects, we employed the experimental autoimmune encephalomyelitis (EAE) mouse model to establish NMOSD animal models. This was achieved by injecting NMO‐IgG into aged mice (11 months old) or using NMO‐IgG along with complement injection and microbubble‐enhanced low‐frequency ultrasound (MELFUS) techniques in young mice (7 weeks old). We assessed the impact of GV‐971 on incidence rate, clinical scores, body weight, and survival, with methylprednisolone serving as a positive control. In NMOSD models of young mice, we analyzed spinal cord samples through H&E staining, immunohistochemistry, and Luxol Fast Blue staining. Fecal samples collected at different time points underwent 16S rRNA gene sequencing, while plasma samples were analyzed using cytokine array and untargeted metabolomics analysis.

**Results:**

Our findings indicated that GV‐971 significantly reduced the incidence of NMOSD, alleviated symptoms, and prolonged survival in NMOSD mouse models. The NMOSD model exhibited substantial neuroinflammation and injury, accompanied by imbalances in gut microbiota, peripheral inflammation, and metabolic disorders, suggesting a potentially vicious cycle that accelerates disease pathogenesis. Notably, GV‐971 effectively reduces neuroinflammation and injury, and restores gut microbiota composition, as well as ameliorates peripheral inflammation and metabolic disorders.

**Conclusions:**

GV‐971 attenuates the progression of NMOSD in murine models and reduces neuroinflammation and injury, likely through its effects on remodeling gut microbiota and peripheral inflammation and metabolic disorders.

## Graphical abstract

The NMOSD model demonstrated substantial neuroinflammation and neural injury, accompanied by imbalances in gut microbiota, peripheral inflammation, and metabolic disorders, suggesting a potentially vicious cycle that exacerbates disease pathogenesis. Notably, GV‐971 effectively reduces neuroinflammation and injury, and restores gut microbiota composition, as well as ameliorates peripheral inflammation and metabolic disorders.

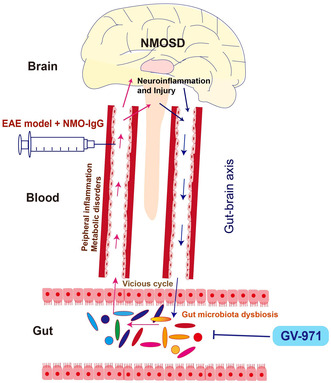

## 1 INTRODUCTION

Neuromyelitis optica spectrum disorders (NMOSD) is an inflammatory demyelinating disease that primarily affects the central nervous system (CNS). Most patients with NMOSD possess specific serum aquaporin‐4 (AQP4) autoantibodies, first identified by Weinshenker in 2007.^1^ This condition predominantly impacts the optic nerve and spinal cord, potentially leading to visual impairment, transverse myelitis, area postrema or brainstem syndrome, and other disabilities.^2^, ^3^ Currently, standard treatment involves immunosuppressive therapies combined with glucocorticoids, which are effective in clinical practice. However, a large portion of patients continue to experience relapses and worsening symptoms despite these treatments. Furthermore, long‐term use of immunosuppressants and glucocorticoids carries potential risks. Given the severe disability, high mortality, and therapeutic challenges associated with NMOSD,^4^, ^5^, ^6^ it is crucial to explore more effective treatments to improve patient outcomes and reduce the risk of recurrence.

The exact causes and mechanisms underlying NMOSD remain inadequately understood. Some reports suggest that changes in peripheral immunity are associated with the development of NMOSD, including factors such as humoral immunity (AQP4 autoantibody and complement),^7^, ^8^ Th17 cells,^9^, ^10^, ^11^, ^12^ CD4T cells,^13^, ^14^, ^15^ CD8T cells,^16^, ^17^ T helper follicular cells,^18^, ^19^ and Tregs.^20^ Recent research has also indicated that an imbalance in gut microbiota composition may play a role in NMOSD. Patients with NMOSD from various populations have shown an increased abundance of certain microbial taxa, such as *Clostridium perfringens*,^21^ *Streptococcus*,^22^, ^23^, ^24^ and *Clostridium bolteae*.^25^ These microbes may have sequences similar to the astrocyte water channel protein AQP4 peptide, potentially triggering Th17 cell activation and disrupting the balance between Th17 cells and Treg cells.^26^ This disturbance in the gut microbiome can lead to intestinal barrier dysfunction^23^ and metabolic pathway alterations,^22^, ^24^, ^26^ resulting in peripheral inflammation and immune responses that may contribute to the breakdown of the blood–brain barrier (BBB) and the infiltration of immune cells into the CNS.^27^, ^28^ AQP4‐IgG, which is produced either in the peripheral circulation or intrathecally, plays a critical role in NMOSD. This antibody damages astrocytes and oligodendrocytes through complement activation and inflammatory infiltration,^29^ with microbial metabolites potentially implicated in this process.^30^, ^31^ These findings open new avenues for potential therapies targeting the gut microbiome in NMOSD.

Given the growing recognition of the importance of the gut microbiome in NMOSD, our goal is to identify effective strategies to address this challenging disease. GV‐971, also known as sodium oligomannate, is a novel medication approved in China for treating mild‐to‐moderate Alzheimer's disease (AD) by targeting the gut‐brain axis.^32^, ^33^, ^34^ In a previous study, we observed that GV‐971 could inhibit pro‐inflammatory Th1 cells by modulating the gut microbiome, reducing Th1 cell infiltration, and mitigating microglial activation, thus alleviating central neuroinflammation in a transgenic AD mouse model.^35^ After oral administration, most of the ingested GV‐971 remains in the gut, but some can penetrate the brain.^36^ This allows GV‐971 to directly inhibit the formation of Aβ fibrils and disrupt preexisting fibrils into non‐toxic monomers,^36^, ^37^ ultimately reducing Aβ deposition in the brain.^35^, ^38^, ^39^, ^40^, ^41^ Additionally, GV‐971 has demonstrated the ability to reduce α‐synuclein aggregation and associated pathology in a Parkinson's disease model.^42^ Considering the potential benefits of GV‐971 in regulating the gut microbiome and its anti‐inflammatory properties, we are exploring the possibility of using GV‐971 in animal models of NMOSD to determine if this unique medication could be beneficial for treating this condition.

## 2 MATERIALS AND METHODS

### 2.1 Neuromyelitis optica spectrum disease model in aged mice

We conducted experiments using 11‐month‐old female C57BL/6 mice obtained from Zhejiang Vital River Laboratory Animal Technology Co., Ltd. The mice were housed in a specific pathogen‐free (SPF) animal room at PharmaLegacy Laboratories (Shanghai) Co., Ltd. All experimental procedures were approved by the Animal Care and Use Committee of Green Valley (Shanghai) Pharmaceuticals Co., Ltd. The collection of plasma from NMOSD patients received approval from the Ethics Committee of Ruijin Hospital Affiliated with Shanghai Jiaotong University School of Medicine (Protocol Number: 2021–375), and informed consent was obtained from the subjects. 2 days prior to injection, we purified human control‐IgG and NMO‐IgG fractions from the plasma of normal individuals and NMOSD patients in the acute relapse phase, respectively, using the MelonTM Gel IgG Purification kit. These patients exhibited high AQP4‐IgG seropositivity (>1:100), determined by a cell‐based assay provided by KingMed Diagnostics company.

To induce experimental autoimmune encephalomyelitis (EAE), we administered myelin oligodendrocyte glycoprotein (MOG) (#51716, GL Biochem (Shanghai), Ltd.) and pertussis toxin (PTX) (#180, List Biological) to mice. On day 0, the mice were randomly assigned to different groups (*n* = 10). After anesthetizing the mice with isoflurane, we subcutaneously injected an emulsion containing 2 mg/mL MOG_35‐55_ and 4 mg/mL complete Freund's adjuvant (CFA) in equal volumes (10 μL each), which had been homogenized for 1 h in a 40 mL brown glass vial. Simultaneously, we intraperitoneally injected 200 μL (200 ng) of a diluted PTX solution. After 48 h, the mice received a second PTX injection. The normal group did not undergo any immunization. Following EAE induction, the mice were intraperitoneally injected with NMO‐IgG antibody (2 mg per mouse) on days 13, 14, 18, and 19 to trigger the onset of NMOSD. The experiment concluded 28 days after the last injection. The NMO control group received IgG obtained from healthy individuals.

Oral administration of GV‐971 at a dose of 200 mg/kg began on the day of immunization and continued until the end of the experiment. Methylprednisolone, used as a positive control, was administered at a dose of 5 mg/kg. We monitored the mice's body weight three times a week from day 0 until the onset of symptoms, typically around day 12. From day 12 onward, we assessed disease symptoms and recorded the clinical score daily using a 5‐point scale, in accordance with criteria established by Hooke Laboratories (Hooke – Contract Research – Experimental autoimmune encephalomyelitis (EAE) – Mouse EAE scoring (hookelabs.com)). Additionally, we recorded the survival time of the mice.

### 2.2 Neuromyelitis optica spectrum disease model in young mice

All experimental procedures were approved by the Animal Care and Use Committee of Green Valley (Shanghai) Pharmaceuticals Co., Ltd. The mice were housed in the SPF class animal room at PharmaLegacy Laboratories (Shanghai) Co., Ltd. Seven‐week‐old female C57BL/6 mice were purchased from Shanghai Lingchang Biological Technology Co., Ltd. We purified human NMO‐IgG fractions from the plasma of NMOSD patients with high AQP4‐IgG seropositivity as previously described.

To establish the EAE model in young mice, we followed a published protocol.^43^ Briefly, on day 0, the mice were randomly assigned to different groups (*n* = 5). After anesthetizing the mice with isoflurane, we subcutaneously injected an emulsion containing 2 mg/mL MOG_35‐55_ and 4 mg/mL complete Freund's adjuvant (CFA) in equal volumes (10 μL each), which had been homogenized for 1 h in a 40 mL brown glass vial. Simultaneously, we intraperitoneally injected 200 μL (200 ng) of a diluted PTX solution. After 48 h, the mice received a second PTX injection. The normal group did not undergo any immunization. Following EAE induction, on the 19th day post‐immunization, in the NMO‐IgG (microbubble‐enhanced low‐frequency ultrasound, MELFUS) group, we injected a microbubble suspension through the tail vein at a dosage of 8 μL/g of body weight. After 20 s, we applied transcranial sonication using an ultrasonic system configured with a frequency of 1 MHz, a focal length of 2.5 cm, and a total radiation duration of 1 min (Wuxi Haiying company). Subsequently, we intravenously administered 100 μg of NMO‐IgG and 100 μL of complement (derived from healthy serum) to the model mice. The NMO‐IgG group received only NMO‐IgG and complement treatment. The preparation of complement followed a previously outlined protocol.^44^

Oral administration of GV‐971 (200 mg/kg) was started on the day of immunization and continued until the end of the experiment. Body weight was recorded three times a week from day 0 to onset (around day 12) and daily from onset to the end of the experiment. Disease symptoms were recorded, and clinical score was evaluated daily using a 5‐point scale from day 12 onwards. Fecal samples were collected on days 0 (baseline), 20 and 35. Plasma and spinal cord samples were collected after sacrifice (*n* = 5).

### 2.3 Hematoxylin and eosin (H&E) staining

After the mice were euthanized, the hearts of the mice were perfused, and the spinal cord was taken and fixed with 4% paraformaldehyde at room temperature for 24 h. The tissue was dehydrated by full immersion and embedded in molten paraffin. Using a leica RM2245, we cut the paraffin tissue blocks into 4 μm thick sections. The sections were baked, dewaxed, cooled to room temperature, and then treated with hematoxylin and eosin staining (solaria G1120). The stained sections were sealed and observed under a microscope (Hamamatsu Nanozoomer2.0HT).

### 2.4 Immunohistochemistry and Luxol Fast Blue staining

Spinal cord sections prepared for immunohistochemical staining were processed using a Leica Mystand. After baking and dewaxing the slices, heat‐induced epitope retrieval was performed using epitope retrieval solution2 (Leica AR9640). Rabbit polyclonal antibodies were used to detect the expression of IBA1 (#019–19,741, 1:500, Wako Chemicals), CD80 (#ab254579; 1:500, Abcam) and CD206 (#ab64693; 1:1000, Abcam), respectively, and stained with Bond™ Polymer Refine Detection (Leica DS9800). After staining, the sections were sealed and observed under a microscope (Hamamatsu Nanozoomer2.0HT). The relative expression (brown intensity) of IBA1 was analyzed by Image J bundled with 64‐bit Java 1.8.0_172. The positive number of CD80 and CD206 was analyzed by QuPath‐0.4.3.Ink.

For Luxol Fast Blue (LFB) staining, baking, and dewaxing the spinal cord sections, immerse the slices in LFB solution and incubate at 60°C for 16 h. After incubation, rinse off the excess stain with 95% alcohol. Then, perform bluing for 5 s using a 0.05% Lithium Carbonate solution, followed by differentiation with ethanol for 1 min. Finally, proceed with dehydration and mounting before performing microscopic examination and scoring. After staining, the sections were sealed and observed under a microscope (ECLIPSE Ci‐L, Nikon). Histopathology scoring criteria of LFB: 0, normal; 1, scattered vacuolation in either gray or white matter of the spinal cord; 2, aggregated vacuolation in either gray or white matter of the spinal cord; 3, aggregated vacuolation in both gray and white matter of the spinal cord; 4, diffuse vacuolation in both gray and white matter; 5, extensive vacuolation of the entire spinal cord.

### 2.5 16S rRNA gene sequencing

All fecal samples were frozen at −80°C. Microbial genomic DNA was extracted from fecal samples using the E.Z.N.A.® soil DNA Kit (Omega Bio‐tek, Norcross, GA, U.S.) according to the manufacturer's protocols.^45^, ^46^ The concentration and purity of the extracted DNA was determined with TBS‐380 and NanoDrop2000, respectively. DNA extract quality was checked on a 1% agarose gel. The V3‐V4 hypervariable regions of the bacterial 16S rRNA gene were amplified with primers 338F (5′‐ACTCCTACGGGAGGCAGCA‐3′) and 806R (5′‐GGACTACHVGGGTWTCTAAT‐3′) by a thermocycler PCR system (GeneAmp 9700, ABI, USA).

PCR reactions were conducted using the following program: 3 min of denaturation at 95°C, 23 cycles of 30 s at 95°C, 30 s for annealing at 58°C, and 30 s for elongation at 72°C, and a final extension at 72°C for 5 min. PCR reactions were performed in triplicate in a 25 μL mixture containing 12.5 μL of 2 × KAPA HiFi HotStart ReadyMix, 5 μL of each primer (1 μM), and 10 ng of template DNA. The resulting PCR products were extracted from a 2% agarose gel and further purified using the AxyPrep DNA Gel Extraction Kit (Axygen Biosciences, Union City, CA, USA) and quantified using Qubit™ 4 Fluorometer (Invitrogen, USA) according to the manufacturer's protocol. Purified amplicons were pooled in equimolar and paired end sequenced (2 × 300) on an Illumina MiSeq™Dx platform (Illumina, San Diego, USA) according to the standard protocols by Green Valley (Shanghai, China).

### 2.6 Processing of 16S rRNA gene sequencing data and analysis

High‐throughput sequencing of 16S rRNA data was analyzed according to the workflow of Ewels PA et al.^47^ Firstly, filtering and trimming of fastq files were performed using Cutadapt.^48^ Secondly, the Silva (v132) 16S rRNA database, based on the naive Bayes classifier, was used as the training set to generate amplicon sequencing variants (ASVs). Then, the sequence ends were merged, chimera removed, and a sequence table produced.

Data analysis was primarily conducted in R 4.1.2, utilizing the phyloseq package (https://joey711.github.io/phyloseq version 1.36.0) and the Microbiome package (https://microbiome.github.io/ version 1.14.0). Alpha diversity metrics, including the Shannon index, were used to describe the richness and evenness of the microbiome in an ecological community. Rarefaction was first performed on raw count using the *rarefy_even_depth* function in the *phyloseq* package, and then shannon diversity was calculated using the *alpha* function in the *microbiome* package. Violinplots of the Shannon index among the four groups were performed by *ggviolin* function of *ggpubr* package with Wilcoxon Rank Sum test *p*‐values added by the *stat_compare_means* function, both functions are embedded in *ggpubr* packae (https://www.rdocumentation.org/packages/ggpubr/versions/0.4.0 version 0.4.0).

Principal Coordinates Analysis (PCoA) was used to evaluate the relationship between samples, also known as Beta diversity. 16S rRNA gene sequencing abundance was filtered by prevalence (>0.2) and minimum count at ASV level (>5), and the remaining taxa were considered core taxa. The raw abundance of core taxa was converted to relative abundance using the *transform* function in the *microbiome* package. The Bray‐Curtis distance between samples was calculated by *ordinate* function in the *phyloseq* package, and a 2‐D scatterplot representing samples' dissimilarity was plotted using *plot_ordination* function. The significance of difference between sample groups was tested using PERMANOVA with *adonis2* function in the *vegan* package.

DESeq2, a popular differential analysis method for count data, was adapted for 16S rRNA data analysis. Only core taxa with a prevalence greater than 0.2 and a minimum count at the ASV level greater than 5 were selected for differential analysis. Prior to DESeq2 analysis, the raw abundance of core taxa was converted to a DESeq2 dds object by the *phyloseq_to_deseq2* function in the *phyloseq* package. Differential expression calculations at the ASV and genus levels were performed using the *DESeq* function in the DESeq2 package. Results were obtained using the *results* function, and ASVs or genera with significant differences between the two groups were identified with a significance threshold of 0.05. A heatmap representing samples' regularized log transformation expression levels was plotted using the *Heatmap* function in the *ComplexHeatmap* package.

### 2.7 Semi‐quantitative measurement of plasma cytokines

The glass chip used to detect 40 mouse cytokines was purchased from RayBiotech Life, Inc. (Catalog #: GSM‐CYT‐5). The glass chip was taken out of the −20°C refrigerator and left at room temperature for 40 min. The side of the glass chip was marked, and the plastic film on the front side was peeled off before placing the glass chip in a vacuum‐drying oven for 3 h. Each sample well was then filled with 100 μL of blocking solution, and the glass chip was placed on a shaker for 1 h at room temperature for blocking. After decanting the blocking solution, two‐fold mouse plasma was diluted in each well. The glass chip was covered with plastic film and placed in a shaker in a refrigerator at 4°C overnight. The plasma was then decanted, and each well was washed 10 times with 250 μL of Wash Buffer A and 5 times with 250 μL of Wash Buffer B at room temperature. The wells were completely decanted of Wash Buffer B after washing.

1.4 mL of sample diluent was added to each tube of detection antibody, and 80 μL of the diluted detection antibody was added to each well. The glass chip was covered with plastic film on a shaker at room temperature for 2 h. The detection antibody was decanted, and the wells were washed 10 times with 250 μL of Wash Buffer A and 5 times with 250 μL of Wash Buffer B at room temperature. Each well was then completely decanted of Wash Buffer B. Next, 1.4 mL of sample diluent was added to each tube of Cy3 equivalent dye‐conjugated streptavidin, and 80 μL of the diluted Cy3 equivalent dye‐conjugated streptavidin was added to each well. The glass chip was covered with aluminum foil and placed on a shaker at room temperature for 1 h. The dye‐conjugated streptavidin was decanted, and each well was washed 10 times with 250 μL of Wash Buffer A and 5 times with 250 μL of Wash Buffer B at room temperature. Finally, the glass chip was rinsed with ddH_2_O, and water droplets were removed with dust‐free paper without touching the surface of the glass chip. Signals were visualized using the InnnScan 710 Microarray Scanner, and the fluorescence intensity data extraction was done using the GAL file along with microarray analysis software.

Principal component analysis (PCA) of cytokine samples was computed using the pca function (centered, scaled) in the PCAtools package (https://github.com/kevinblighe/PCAtools version 2.4.0) after log transformation. The PCA scatterplot was plotted using the biplot (encircle = TURE) function in the PCAtools package.

### 2.8 Untargeted metabolomics

An aliquot of 60 μL of mouse plasma was mixed with 200 μL of acetonitrile and vortexed for 10 min. The resulting mixture was then centrifuged at 14,000 rpm for 10 min at 4°C. The supernatant was further centrifuged, concentrated until completely dry, and then reconstituted in 120 μL of acetonitrile/water (2:8, v/v). Subsequently, 100 μL of the reconstituted sample was transferred to the autosampler for subsequent analysis, and the remaining portion was combined to create a pooled quality control sample.

For the untargeted metabolomics analysis, an Acquity ultra‐performance liquid chromatography system (Waters, Milford, MA, USA) coupled with a Triple TOF 6600 mass spectrometer system (SCIEX, Framingham, USA) was used in both positive and negative electrospray ionization (ESI) modes. The mass spectrometer parameters were set as follows: curtain gas (CUR) at 35, ion source gas 1 (GAS 1) at 50, ion source gas 2 (GAS 2) at 50, ion spray voltage floating (ISVF) at 5500/−4500 V, ion source temperature (TEM) at 500°C, declustering potential (DP) at 80/−80 V, and collision energy (CE) at 10/−10 eV. Information‐dependent MS/MS acquisition (IDA) was performed on the top 10 most intense precursor ions with a collision energy (CE) set at 35 ± 15 eV. The time‐of‐flight mass analyzer had a scanning range of m/z 50–1000. The injection volume was 5.0 μL, the sample manager temperature was maintained at 8°C, the flow rate was set at 0.35 mL/min, and the column temperature was regulated at 50°C.

In ESI positive mode, chromatographic separation was carried out on a Waters Acquity BEH C8 column (100 mm × 2.1 mm, 1.7 μm). The mobile phases A and B consisted of 0.1% (v/v) formic acid aqueous solution and 0.1% (v/v) formic acid acetonitrile solution, respectively. The elution gradient started with 5% B, held for 1 min, linearly increased to 100% B at 24 min, maintained for 4 min, then reverted to 5% B within 0.1 min and held for 2 min for system stabilization. In ESI negative mode, chromatographic separation was performed on a Waters Acquity HSS T3 column (100 mm × 2.1 mm, 1.8 μm). Mobile phases A and B consisted of 10 mM ammonium acetate aqueous solution containing 0.04% ammonia and 10 mM ammonium acetate with 0.04% ammonia in 95% methanol in water, respectively. The elution gradient started with 2% B, maintained for 1 min, linearly increased to 100% B at 18 min, kept for 4 min, then decreased to 2% B within 0.1 min and held for 3 min for system equilibration.

Raw data were collected and processed using Analyst TF 1.8.1 and MarkerView 1.3.1 software. MetaboAnalyst 6.0 software was employed for statistical analysis, including Chemometrics analysis (partial least squares‐discriminant analysis (PLS‐DA) and sparse partial least squares‐discriminant analysis (sPLS‐DA)) and Univariate analysis (volcano plot), as well as functional enrichment analysis. Under the ESI positive and negative ion detection modes, 3170 and 1108 metabolic features (characterized by unique mass‐to‐charge ratio and chromatographic retention time pairs) were extracted, respectively. Following the removal of 40% of high‐variant metabolic features using the interquartile range filtration method, 1902 and 664 metabolic features were included in the subsequent statistical analysis. Additionally, a logarithmic transformation of the data to the base of 10 was performed, and the data samples were scaled using Auto scaling to standardize the response values of each metabolite to a uniform scale. OSI‐SMMS (version 1.0, Dalian Chem Data Solution Information Technology Co. Ltd.) was utilized for peak annotation following data processing with an in‐house MS/MS database.

## 3 RESULTS

### 3.1 GV‐971 inhibited the onset and progression of NMOSD in aged mice

**FIGURE 1:**
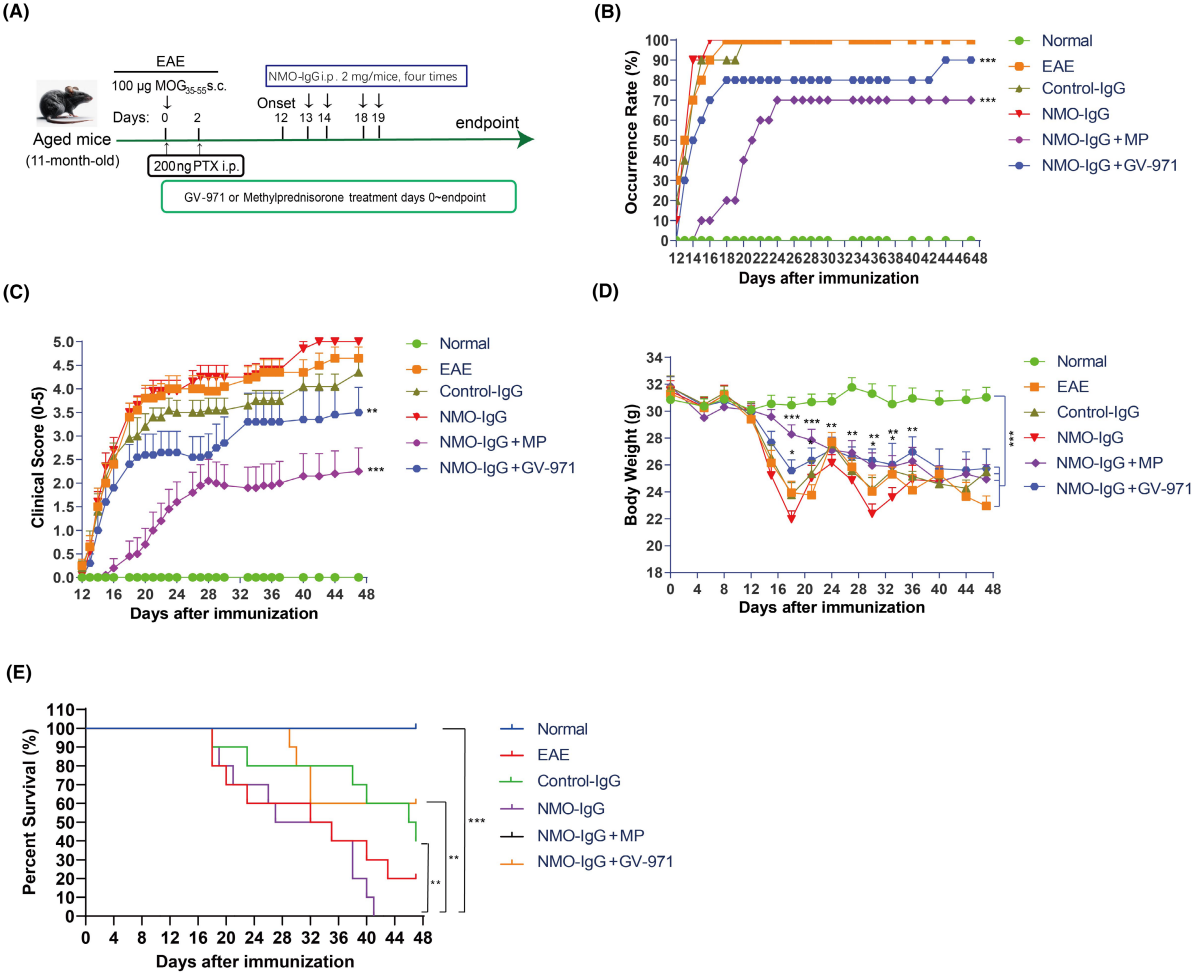
GV‐971 inhibited onset and progression of NMOSD model in aged mice. (A) Overview of study design. (B) GV‐971 or methylprednisolone (MP) decreased incidence rate of NMO‐IgG model of aged mice. (C) GV‐971 or MP reduced clinical score in NMO‐IgG model of aged mice. (D) GV‐971 or MP reduced body weight loss in NMO‐IgG model in aged mice. (E) GV‐971 or MP prolonged survival in NMO‐IgG model of aged mice. Two‐way ANOVA, *p < 0.05, **p < 0.01, ***p < 0.001, as compared to the NMO‐IgG group or as indicated.

We developed a novel NMOSD animal model in aged mice using the experimental autoimmune encephalomyelitis (EAE) model and injected human neuromyelitis optica immunoglobulin G antibody (NMO‐IgG). This model was compared with the EAE model and the EAE model injected with human control‐IgG. Additionally, we assessed the therapeutic effects of GV‐971 on the NMO‐IgG model, with methylprednisolone (MP) used as a positive control (Figure 1A). We observed that the occurrence rate in the EAE model group, control‐IgG group, and NMO‐IgG model group all reached 100%, with no significant differences among them (Figure 1B). However, the occurrence rate in the GV‐971‐treated NMO‐IgG group was 70% at day 16 and increased to 90% at the later stage of the experiment, which was significantly lower than that of the NMO‐IgG group (Figure 1B). Besides, the incidence increased slowly after MP treatment, reaching 70% on day 24, with no subsequent increase, which was much lower than the NMO‐IgG group (Figure 1B).

We further observed the impact of GV‐971 on disease symptoms. The clinical scores in the EAE, control‐IgG, and NMO‐IgG groups gradually increased, with no significant differences among them. However, the clinical score for the control‐IgG group tended to decrease compared to the EAE group. In contrast, the clinical score for the NMO‐IgG group was higher than the EAE group in the later stages of the experiment (Figure 1C). Clinical scores significantly decreased in the GV‐971 or MP‐treated groups compared to the NMO‐IgG group. GV‐971 was slightly less effective than MP, but there was no significant difference between the two treatment groups (Figure 1C). While weight loss was observed in the EAE, control‐IgG, and NMO‐IgG groups, with no significant difference among the three model groups (Figure 1D), this weight loss was slowed in the GV‐971 or MP‐treated groups compared to the NMO‐IgG group. MP had a more pronounced effect, but the difference between the two treatment groups was not significant at later stages of the experiment (Figure 1D).

We also examined the survival rate of mice. Although there were no significant differences among the three model groups, the NMO‐IgG group showed a trend of shorter survival, and the control‐IgG group had a trend of longer survival compared to the EAE model. Interestingly, the survival time was significantly shorter in the NMO‐IgG group than in the control‐IgG group (Figure 1E), further confirming the establishment of the NMOSD disease model in aged mice. Importantly, we observed that GV‐971 significantly prolonged the survival time of NMO‐IgG mice, although not as dramatically as the MP‐treated group (Figure 1E).

### 3.2 GV‐971 suppressed the onset and progression in an NMOSD model of young mice

**FIGURE 2:**
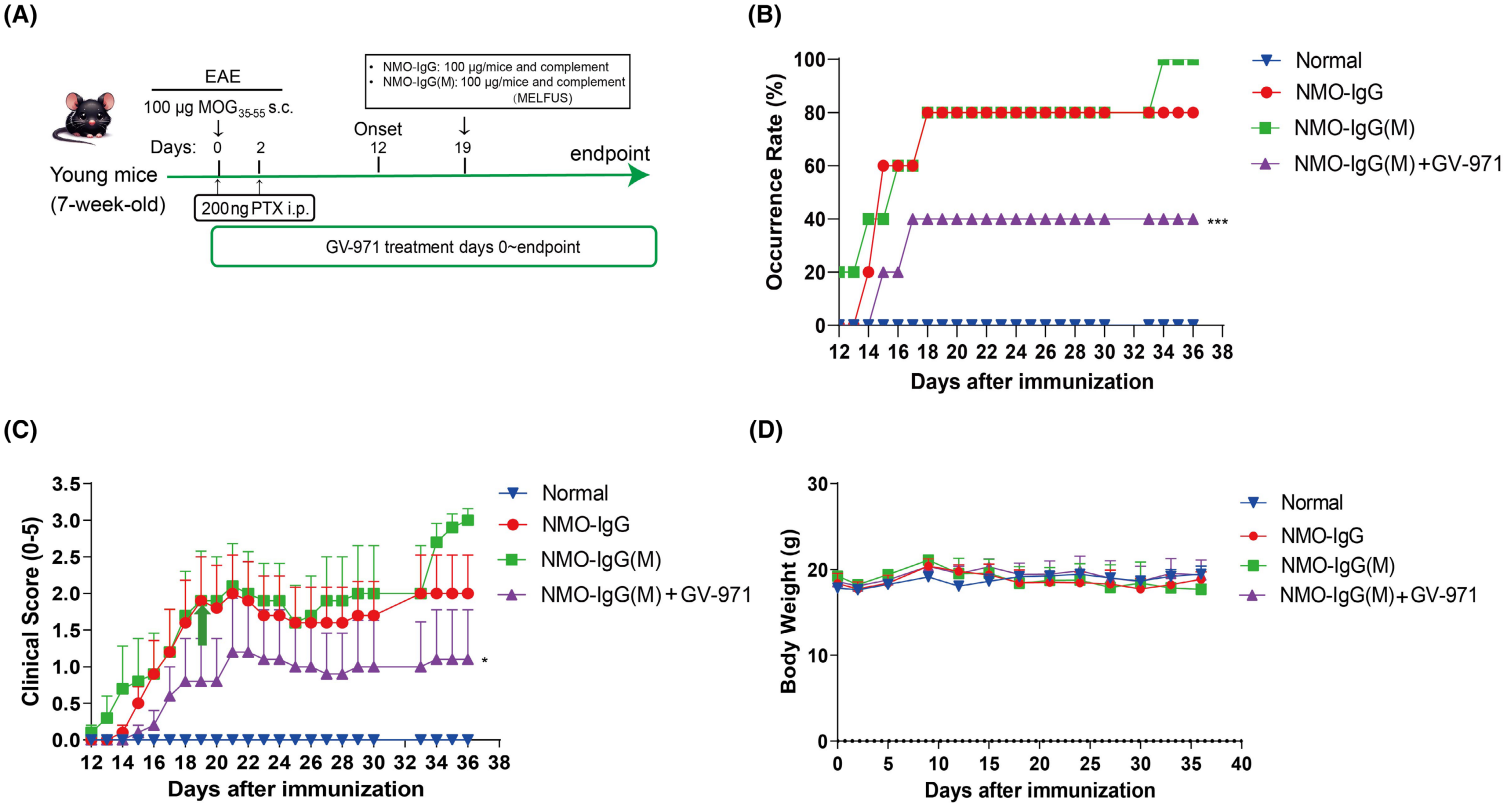
GV‐971 inhibited onset and progression of NMOSD model in young mice. (A) Overview of study design. (B) GV‐971 decreased incidence rate of NMO‐IgG(M) model in young mice, the incidence rate is higher in NMO‐IgG(M) group than NMO‐IgG group. (C) GV‐971 relieved symptoms in NMO‐IgG(M) model mice, the clinical score was higher in NMO‐IgG(M) group than NMO‐IgG group. (D) Body weight change of NMOSD model in young mice. Two‐way ANOVA, *p < 0.05, ***p < 0.001, as compared to the NMO‐IgG(M) group.

Building on the above work, we evaluated the therapeutic potential of GV‐971 in a reported NMOSD mouse model.^43^ In this NMOSD model, we utilized young mice that were 7 weeks old. We employed low‐frequency focused ultrasound combined with the microbubble technique (MELFUS) and complement injection in addition to the administration of NMO‐IgG (Figure 2A). MELFUS(M) was used to open the blood–brain barrier, facilitating the entry of antibodies and complement into the central nervous system, thereby promoting injury. Although there was no statistically significant difference between the two model groups, we observed that the young mice in the NMO‐IgG injection group had an 80% occurrence rate, whereas the NMO‐IgG(M) group had a 100% occurrence rate (Figure 2B). The occurrence rate was significantly reduced to 40% in the GV‐971‐treated group compared to the NMO‐IgG(M) group (Figure 2B).

Regarding disease symptoms, although there was no significant difference between the NMO‐IgG and NMO‐IgG(M) groups, we noticed a trend towards increased symptoms in the NMO‐IgG(M) group in the later stages. Intriguingly, GV‐971 treatment significantly alleviated disease symptoms in mice compared to the NMO‐IgG(M) group (Figure 2C), providing further evidence of the therapeutic efficacy of GV‐971 in the NMOSD model. Additionally, mice in both model groups and the GV‐971‐treated group did not exhibit significant changes in body weight (Figure 2D).

### 3.3 GV‐971 alleviated neuroinflammation in NMOSD model mice

**FIGURE 3:**
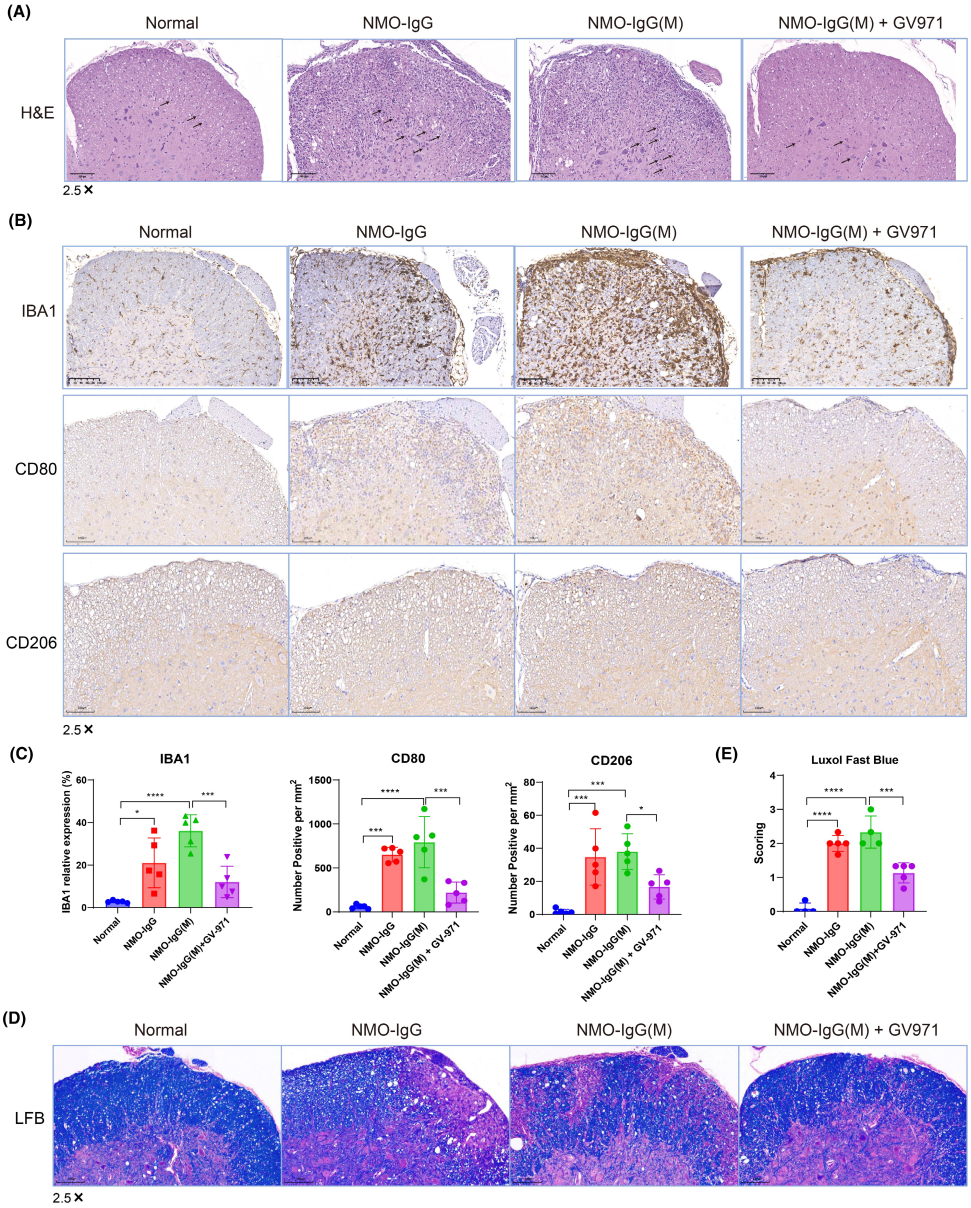
GV‐971 alleviated neuroinflammation and neural damage in NMOSD model mice. (A) Representative H&E staining of spinal cord. Increased immune cell infiltration and vacuole in NMO‐IgG group and more pronounced in NMO‐IgG(M) group, while diminished in NMO‐IgG(M) + GV‐971 group. The magnification is 2.5×, *n* = 5. (B) Representative immunohistochemical staining of IBA1, CD80, and CD206. Increased IBA1, CD80, and CD206 in NMO‐IgG group and more pronounced in NMO‐IgG(M) group, while decreased in NMO‐IgG(M) + GV‐971 group. The magnification is 2.5×. (C) The relative expression of IBA1, CD80 and CD206 of (B). (D) Luxol Fast Blue (LFB) staining of myelin in spinal cord among four groups. (E) The scoring of myelin as determined by LFB in (D). Data presented as mean ± SD. One‐way ANOVA, *p < 0.05, **p < 0.01, ***p < 0.001, ****p < 0.0001, *n* = 5.

In the NMOSD model of aged mice, we were unable to obtain animal tissue samples due to survival concerns. Consequently, we collected spinal cord samples from the NMOSD model of young mice to investigate the pathological changes associated with neuroinflammation and injury. Using the H&E staining method, we observed increased infiltration of immune cells in the spinal cord in both the NMO‐IgG and NMO‐IgG(M) groups, along with multiple intercellular vacuoles compared to the normal group. The GV‐971‐treated group exhibited reduced immune cell infiltration and fewer intercellular vacuoles compared to the NMO‐IgG(M) group (Figure 3A). This suggests that GV‐971 significantly mitigated the inflammatory damage in the spinal cord.

We further assessed neuroinflammation by examining the expression of IBA1, a marker for microglia activation, using immunohistochemistry. The results revealed significantly higher IBA1 expression in the NMO‐IgG group, with even higher levels in the NMO‐IgG(M) group compared to the normal group. Notably, GV‐971 treatment led to a significant reduction in IBA1 expression compared to the NMO‐IgG(M) group (Figure 3B,C). Additionally, we evaluated the expression of CD80 (activation B7‐1 antigen) and CD206 (macrophage mannose receptor 1) in the spinal cord, markers indicating the M1‐type and M2‐type macrophages, respectively. We found that the expression of CD80 and CD206 was significantly increased in both the NMO‐IgG and NMO‐IgG(M) groups. However, treatment with GV‐971 significantly reduced the expression of these markers, suggesting that GV‐971 can significantly reduce the infiltration of M1‐like and M2‐like macrophages induced in the NMO‐IgG(M) group (Figure 3B,C).

Furthermore, we employed the Luxol Fast Blue method to detect spinal cord injury, where blue indicates the presence of myelin, and non‐blue or vacuolated areas indicate demyelination or areas lacking myelin. The severity of the injury was then scored accordingly. The results showed that compared to the normal group, spinal cord injury was significantly increased in the NMO‐IgG and NMO‐IgG(M) groups. However, treatment with GV‐971 significantly reduced spinal cord injury compared to the NMO‐IgG(M) group (Figure 3D,E).

Collectively, these results indicate that neuroinflammation and injury were significantly increased in the NMO‐IgG and NMO‐IgG(M) groups. Treatment with GV‐971 significantly reduced the neuroinflammation and injury caused by the modeling.

### 3.4 GV‐971 remodeled the gut microbiota composition in NMOSD model mice

**FIGURE 4:**
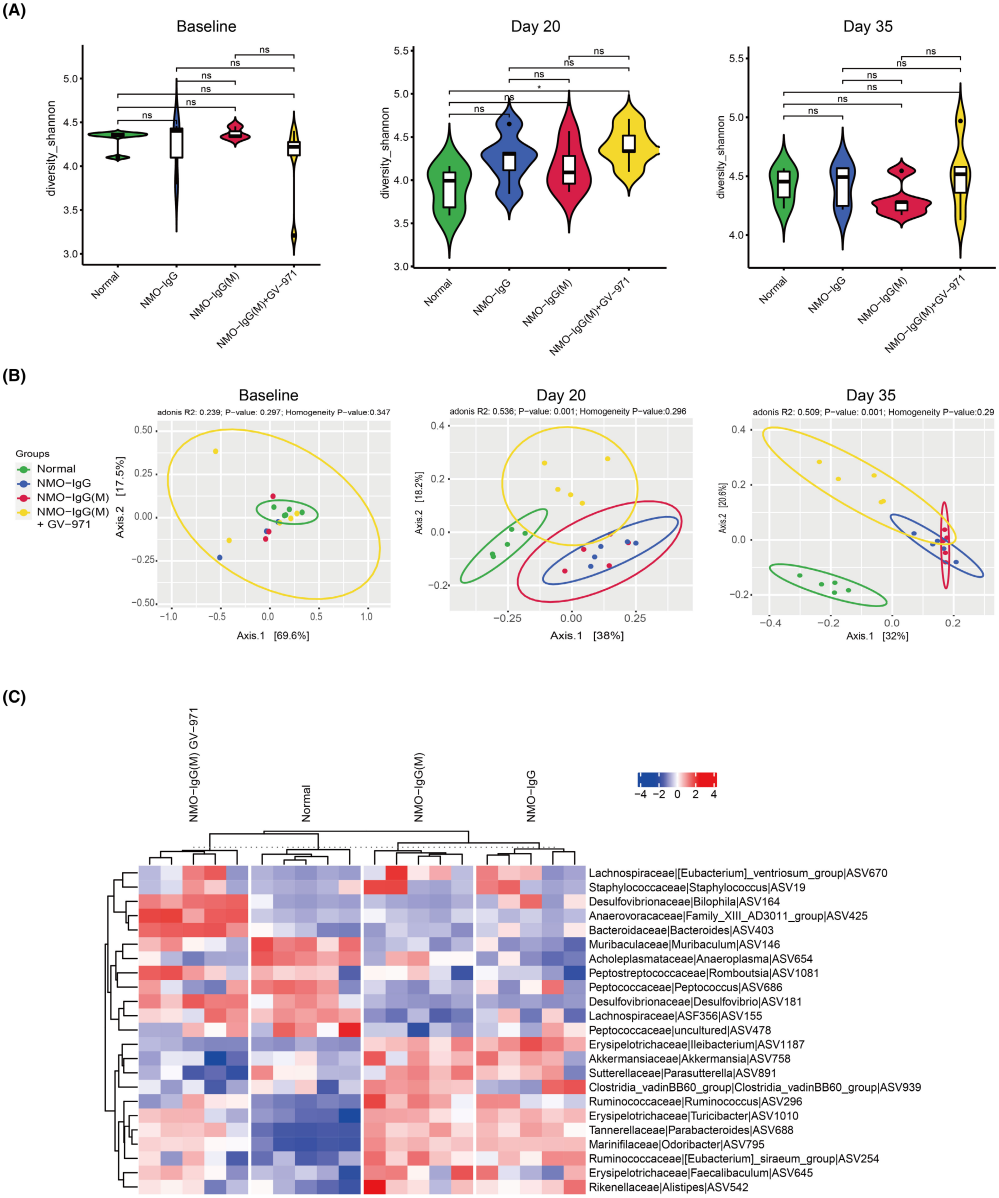
GV‐971 remodeled gut microbiota composition in NMOSD model mice. (A) Shannon diversity of gut microbiota in each group; ns: No significant difference; *p < 0.05. (B) Principal co‐ordinates analysis (PCoA) of gut microbiota on baseline, day 20, and day 35; Dramatic change in gut microbial composition in NMO‐IgG group and NMO‐IgG(M) group; Recovery in NMO‐IgG(M) plus GV‐971 group in PC1 dimension on days 20 and 35. (C) Differential analysis of gut microbial composition on day 35 (endpoint). Heatmap represents samples' regularized Log transformation expression level; *p* < 0.05 as significance threshold; ASV or genus with significant differences identified.

To investigate whether GV‐971 reduces neuroinflammation by influencing the gut microbiota, we collected fecal samples at various time points in the NMOSD model of young mice, including before modeling (baseline), during peak onset (day 20), and at the endpoint (day 35). We then conducted an analysis of the structural changes in the gut microbiota using 16S RNA gene sequencing. At baseline, the Shannon diversity of the gut microbiota did not show significant differences among the groups. However, at day 20, there was a noteworthy increase in the GV‐971‐treated group compared to the normal group, while there were no significant differences at the endpoint among the groups (Figure 4A). This suggests that the modeling and GV‐971 treatment did not have a profound impact on the diversity of the gut microbiota.

We further employed Principal Co‐ordinates Analysis (PCoA) to compare the composition of the gut microbiota among the groups. We observed no significant differences in the composition of the gut microbiota between the groups at baseline. However, on day 20, there were dramatic changes in the NMO‐IgG and NMO‐IgG(M) groups, with similar patterns observed between these two groups (Figure 4B). This indicates that both the EAE modeling and NMO‐IgG injection affected the composition of the gut microbiota. Intriguingly, GV‐971 was found to reverse these changes in the gut microbiota, as demonstrated on both day 20 and day 35 (Figure 4B). Additionally, we noticed that the differential gut microbes were similar in the NMO‐IgG and NMO‐IgG(M) groups, whereas in the GV‐971‐treated group, the differential gut microbes were more aligned with the normal group (Figure 4C). Collectively, these results indicate that GV‐971 has the capacity to remodel the composition of the gut microbiota, which were parallel with the alleviation of neuroinflammation.

### 3.5 GV‐971 mitigated peripheral inflammation and reversed metabolic disorder in NMOSD model mice

**FIGURE 5:**
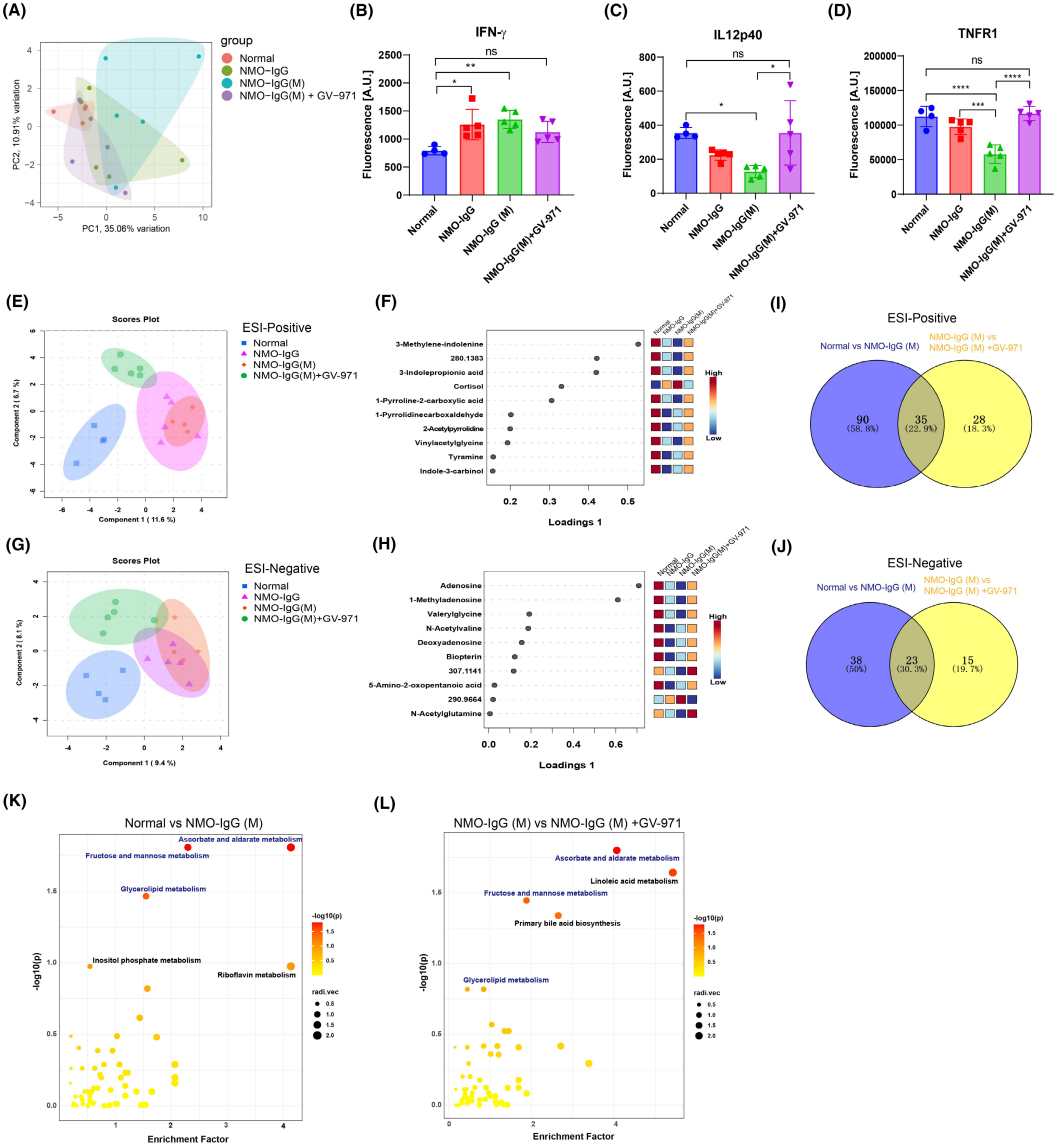
Effect of GV‐971 on plasma cytokines and metabolites in NMOSD model mice. (A) Principal component analysis (PCA) of plasma 40 mouse cytokines across 4 groups: Normal control, NMO‐IgG, NMO‐IgG(M), and NMO‐IgG(M) + GV971. Raw cytokines abundance log transformed, centered, and scaled. (B–D) Fluorescence intensity (a.u.) of plasma cytokines in each group. Data presented as Mean ± S.E.M. One‐way ANOVA, *p < 0.05, **p < 0.01, ***p < 0.001, ^****^ *p* < 0.0001. (E) sPLS‐DA analysis of ESI‐positive metabolites among four groups. (F) The top 10 discriminative metabolites among four groups in ESI‐positive mode of (E). (G) sPLS‐DA analysis of ESI‐negative metabolites among four groups. (H) The top 10 discriminative metabolites among four groups in ESI‐negative mode of (G). (I) Venny plot of differential metabolites between NMO‐IgG(M) vs. normal and NMO‐IgG(M) + GV‐971 vs. NMO‐IgG(M) in ESI positive mode, the number and percentage of differential metabolites were shown (Fold chage≥2, *p* < 0.05). (J) Venny plot of differential metabolites between NMO‐IgG(M) vs. normal and NMO‐IgG(M) + GV‐971 vs. NMO‐IgG(M) in ESI negative mode, the number and percentage of differential metabolites were shown (Fold chage ≥ 2, *p* < 0.05). (K) KEGG enriched pathways of differential metabolites between NMO‐IgG(M) and normal groups. (L) KEGG enriched pathways of differential metabolites between NMO‐IgG(M) + GV‐971 and NMO‐IgG(M) groups.

We proceeded to explore whether GV‐971 could modulate peripheral inflammation. 40 inflammation‐related cytokines were examined in the plasma of NMOSD model mice using a cytokine array. Our findings revealed a notable shift in the overall expression of plasma cytokines in the NMO‐IgG group, with a more pronounced shift in the NMO‐IgG(M) group. However, in the GV‐971‐treated group, the overall expression of plasma cytokines was reversed and resembled that of the normal group (Figure 5A). Notably, we found three cytokines that showed significant alterations in the NMO‐IgG(M) group: an increase in IFN‐γ and a decrease in IL12p40 and TNFR1. GV‐971 treatment successfully restored these cytokine levels to their baseline (Figure 5B–D). Additionally, many cytokines exhibited a tendency to change which were reversed by GV‐971, including the proinflammatory IL‐1β, IL‐6, TNF‐α (Figure S1A–C), anti‐inflammatory IL‐10 (Figure S1D), myeloid differentiation‐related GM‐CSF and IL‐3 (Figure S1E,F), lymphocyte proliferation‐related IL‐2, IL‐5, IL‐7, and IL‐21 (Figure S1G–J), and chemotaxis‐related KC, TARC, Eotaxin‐2, and MCP‐1 (Figure S1K–N). These findings suggest that GV‐971 has the capacity to restore plasma cytokine levels and alleviate peripheral inflammation.

Given the important role of metabolites in mediating the gut microbiota‐brain axis, we explored changes in plasma metabolites using untargeted metabolomics. sPLS‐DA was used to analyze the overall pattern of metabolites in ESI‐positive mode, we found that the NMO‐IgG and the NMO‐IgG(M) groups were distant from the normal group in the component 1 dimension, whereas the NMO‐IgG(M) + GV‐971 group was closer to the normal group (Figure 5E), suggesting that GV‐971 reverses the changes in metabolites of the NMO‐IgG(M) group. By displaying the top 10 metabolites of component 1 in ESI‐positive mode, we found that cortisol levels were higher in the NMO‐IgG and NMO‐IgG(M) groups but decreased in the GV‐971‐treated group. Other metabolites showed consistent changes: they were lower in the NMO‐IgG and NMO‐IgG(M) groups but increased in the GV‐971‐treated group, including 3‐methylene‐indolenine, 3‐indolepropionic acid, 1‐pyrroline‐2‐carboxylic acid, 1‐pyrrolidinecarboxaldehyde, 2‐acetylpyrrolidine, vinylacetylglycine, tyramine, and indole‐3‐carbinol (Figure 5F). Similarly, sPLS‐DA analysis of the ESI‐negative mode metabolites also showed that the NMO‐IgG and the NMO‐IgG(M) groups were distant from the normal group in the component 1 dimension, while the NMO‐IgG(M) + GV‐971 group was closer to the normal group (Figure 5G). By analyzing the top 10 metabolites of component 1 in ESI‐negative mode, we found that most metabolites exhibited consistent changes: they were lower in the NMO‐IgG and NMO‐IgG(M) groups but increased in the GV‐971‐treated group, including adenosine, 1‐methyladenosine, valerylglycine, N‐acetylvaline, deoxyadenosine, biopterin, and 5‐amino‐2‐oxopentanoic acid (Figure 5H). These results indicated that GV‐971 treatment reverses the changes in plasma metabolites observed in the model group.

Using PLS‐DA to analyze the major distinguishing metabolites between different groups, we found that in the ESI‐positive mode, the top 40 distinguishing metabolites between the NMO‐IgG(M) group and the normal group overlapped with those between the NMO‐IgG(M) + GV‐971 group and the NMO‐IgG(M) group, including cortisol, 3‐indolepropionic acid, 3‐methylene‐indolenine, and 1,24,25‐trihydroxyvitamin D3. These metabolites' changes in model group were reversed by GV‐971 treatment (Figure S2A,B). Similarly, in the ESI‐negative mode, the top 40 distinguishing metabolites between the NMO‐IgG(M) group and the normal group also overlapped with those between the NMO‐IgG(M) + GV‐971 group and the NMO‐IgG(M) group, including 1‐methyladenosine, biopterin, deoxyadenosine, and adenosine. These metabolites' changes in model group were also reversed by GV‐971 treatment (Figure S2C,D). These data indicate that GV‐971 has a reversing effect on the changes in plasma metabolites observed in the model group.

Next, we analyzed the significantly altered metabolites between different groups and found many significantly changed metabolites between the NMO‐IgG(M) and normal groups in both ESI‐positive and ESI‐negative modes (Figure S2E,F). Similarly, we found many significantly altered metabolites between the NMO‐IgG(M) + GV‐971 and NMO‐IgG(M) groups in both ESI‐positive and ESI‐negative modes (Figure S2G,H). Notably, we also found that a proportion of these differential metabolites overlapped between the aforementioned groups in both ESI‐positive and ESI‐negative modes (Figure 5I,J, Tables S1 and S2). Interestingly, most of these overlapping metabolites showed a reversal of the changes by GV‐971 treatment (Tables S1 and S2), further indicating the remodeling effect of GV‐971 on the plasma metabolites.

Furthermore, KEGG pathway enrichment analysis of the differential metabolites between the NMO‐IgG(M) and normal groups revealed that these pathways mainly involved ascorbate and aldarate metabolism, fructose and mannose metabolism, glycerolipid metabolism, inositol phosphate metabolism, and riboflavin metabolism (Figure 5K). KEGG pathway enrichment analysis of the differential metabolites between the NMO‐IgG (M) + GV‐971 and NMO‐IgG(M) groups revealed that these pathways mainly involved ascorbate and aldarate metabolism, fructose and mannose metabolism, glycerolipid metabolism, linoleic acid metabolism, and primary bile acid biosynthesis (Figure 5L). Interestingly, the enriched metabolic pathways after GV‐971 treatment largely overlapped with those of the model group (Table S3), further suggesting the remodeling effect of GV‐971 on the altered metabolites. Collectively, these data indicate that GV‐971 has the effect of restoring metabolic disorders in the model group.

## 4 DISCUSSION

In the current study, we employed the EAE mouse model as a basis to develop NMOSD animal models in both aged and young mice. Our observations indicated a higher disease incidence and elevated clinical scores in the NMOSD models compared to the EAE model, suggesting more severe symptoms in the NMOSD models. Notably, this is the first study to establish an NMOSD model in aged mice. Our findings revealed that GV‐971 treatment resulted in a significant reduction in disease incidence and clinical symptoms, as well as an extension of the survival time for NMOSD‐afflicted aged mice. Although GV‐971's impact may not be as pronounced as that of MP in this model, its exploration as a treatment option for NMOSD holds considerable therapeutic promise, particularly for long‐term maintenance therapy following immunosuppressant and glucocorticoid treatments.

Furthermore, we demonstrated that GV‐971 significantly reduced disease incidence and clinical scores in the NMOSD model featuring young mice. The increased neuroinflammation and injury in the spinal cords of both the NMO‐IgG and NMO‐IgG(M) groups were found, as evidenced by H&E staining, IBA1, CD80, and CD206 staining, as well as Luxol Fast Blue staining. Importantly, GV‐971 treatment consistently alleviated neuroinflammation and injury. These results underscore the potential of GV‐971 as a viable therapeutic option for NMOSD.

It is noteworthy that we did not actively manipulate gut microbiota during the modeling process, yet we observed significant compositional changes in the gut microbiota within the NMO‐IgG and NMO‐IgG(M) groups. This suggests that the induction of an inflammatory response by NMO‐IgG affects gut microbiota composition. Previous research has indicated that alterations in gut microbiota can provoke peripheral inflammation and even neuroinflammation.^35^, ^49^, ^50^, ^51^, ^52^ Based on these findings, we hypothesized that changes in gut microbiota composition in the NMOSD model may further contribute to peripheral inflammation, metabolic disorders, and neuroinflammation. Consistent with this hypothesis, our data showed notable shifts in the inflammatory and metabolic profiles of peripheral blood in the NMO‐IgG and NMO‐IgG(M) groups compared to the normal group. Collectively, these findings suggest that NMOSD modeling leads to significant neuroinflammation and concurrent disruptions in gut microbiota composition and peripheral system. Altered gut microbiota composition and peripheral disorders may exacerbate neuroinflammation, creating a vicious cycle. Therefore, therapeutic agents capable of reshaping gut microbiota composition and mitigating peripheral disorders hold promise for treating this condition.

GV‐971, a drug currently used for mild‐to‐moderate Alzheimer's disease (AD) in China, functions by reshaping gut microbiota to mitigate peripheral inflammation, thereby ameliorating neuroinflammation.^35^ Guided by this principle, we investigated the impact of GV‐971 on gut microbiota composition, peripheral disorders, and neuroinflammation in an NMOSD mouse model. Our findings revealed that GV‐971 significantly remodeled the gut microbiota, reduced peripheral inflammation and metabolic disorder, and alleviated neuroinflammation in the spinal cord. Given that GV‐971 is primarily localized to the intestinal tract after oral administration and exhibits extremely low oral bioavailability in rats (0.6% ~ 1.6%) and dogs (4.5% ~ 9.3%),^36^ we hypothesize that its role in remodeling gut microbiota and peripheral disorders is crucial in mitigating neuroinflammation. Nevertheless, we cannot dismiss the possibility that GV‐971 directly inhibits neuroinflammation, despite its low bioavailability. This potential effect warrants further investigation.

Considering the gut microbiota's intricate composition and the diverse compositional alterations it undergoes in distinct disease models, the genera influenced by GV‐971 in our NMOSD model differ from those observed in previous AD models.^35^ As such, a detailed investigation is warranted to elucidate the specific regulatory mechanism employed by GV‐971 in the future. Nonetheless, a recurring observation is that GV‐971 tends to modify the overall structure of the gut microbiota in different disease models, suggesting that its influence likely operates through a collective regulation of the gut microbiota community rather than targeting individual genera or a single genus.

Regarding plasma cytokine changes, we found that GV‐971 effectively reverses the levels of plasma cytokines altered by the disease model. Notably, IFN‐γ, IL12p40, and TNFR1 exhibited significant changes in the NMO‐IgG(M) group compared to the normal group, which were reversed by GV‐971 treatment. IFN‐γ is a multifaceted cytokine playing a crucial role in macrophage activation, antigen presentation enhancement, Th1 response, and cytotoxic T cell activation.^53^ Overproduction of IFN‐γ can contribute to autoimmune disease pathology, including NMOSD and multiple sclerosis.^54^, ^55^, ^56^ Thus, GV‐971's inhibition of IFN‐γ may reduce the immune response in the NMOSD model. IL‐12p40, a subunit shared by interleukin‐12 (IL‐12) and interleukin‐23 (IL‐23), plays a significant role in inflammatory responses and autoimmunity.^57^, ^58^, ^59^, ^60^ Interestingly, IL‐12 also has neuroprotective effects, reducing early neurodegeneration and sustaining trophic factor release in autoimmune conditions, including those of the CNS.^61^ In our study, IL‐12p40 levels decreased in the NMO‐IgG(M) group but increased after GV‐971 treatment, suggesting its neuroprotective role in this model. TNFR1, a key receptor in the TNF signaling pathway, regulates inflammation and apoptosis.^62^ Elevated plasma levels of soluble TNFR1 can act as a decoy receptor, binding to TNF‐α and modulating its bioavailability and activity.^63^, ^64^ The decrease of TNFR1 in the model group and its increase after GV‐971 treatment suggest it may function as a decoy receptor to antagonize inflammation. Therefore, GV‐971's effects on plasma cytokines indicate its potential to alleviate peripheral inflammation and promote tissue repair, potentially reducing neuroinflammation.

Through untargeted metabolomics analysis of plasma metabolites, we discovered that GV‐971 significantly remodels metabolic alterations induced by the disease model. GV‐971 reversed key metabolites, including indoles (3‐methylene‐indolenine, 3‐indolepropionic acid, indole‐3‐carbinol), pyrrolidine analogs (1‐pyrroline‐2‐carboxylic acid, 1‐pyrrolidinecarboxaldehyde, 2‐acetylpyrrolidine), and adenosine analogs (adenosine, 1‐methyladenosine, deoxyadenosine). Indoles, produced from dietary tryptophan by symbiotic bacteria in the gastrointestinal tract, possess antioxidant, anti‐inflammatory, and neuroprotective functions, such as 3‐indolepropionic acid^65^, ^66^ and indole‐3‐carbinol.^67^ Given that indoles are produced by gut microbiota, their decline in the model group and increase following GV‐971 treatment suggest that GV‐971 promotes the synthesis of indoles by gut microbiota, thereby enhancing anti‐inflammatory, antioxidant, and neuroprotective functions. Pyrrolidines, with anti‐inflammatory and antiviral functions,^68^ which could be metabolized by the gut microbiota, modify their activity.^69^ The reduction of pyrrolidines in the model group and their increase after GV‐971 treatment suggest that GV‐971 enhances the production of these metabolites by remodeling gut microbiota, thereby reducing peripheral inflammation and neuroinflammation. Adenosine, a component of ATP, possesses anti‐inflammatory and neuroprotective functions.^70^ The increase in adenosine analogs following GV‐971 treatment indicates a reduction in inflammation and promotion of neural repair. Additionally, cortisol levels, elevated in the model group, were reduced with GV‐971 treatment, indicating that GV‐971 alleviates the stress response induced by the disease model. Taken together, GV‐971 has a remodeling effect on the metabolic changes in the disease model, and the identified key metabolite alterations associated with gut microbiota function may mediate the anti‐inflammatory and neuroprotective effects of GV‐971, warranting further in‐depth validation.

In conclusion, we successfully established two NMOSD mouse models and demonstrated the therapeutic efficacy of GV‐971 in NMOSD. GV‐971 treatment significantly ameliorated disease symptoms and mitigated neuroinflammation and injury in the spinal cord. Furthermore, we observed simultaneous alterations in gut microbiota composition, peripheral inflammation, and metabolite profiles in the model group, which were reversed by GV‐971 treatment. Our findings suggest that GV‐971's ability to modify gut microbiota composition and peripheral inflammatory and metabolic profiles may be associated with its capacity to suppress neuroinflammation and injury.

However, our study has several limitations. This study primarily evaluated the therapeutic effects of GV‐971 on NMOSD animal models, observing changes in gut microbiota, plasma cytokines, and metabolites that paralleled neuroinflammation and injury, suggesting potential links among these factors. GV‐971 may play a modulatory role in this gut‐brain axis. Nevertheless, the mechanistic study of causal relationships between these factors remains insufficient. Understanding how GV‐971 regulates gut microbiota and reduces peripheral inflammation and metabolites to mediate its suppression of neuroinflammation remains a formidable challenge. Future research endeavors will focus on elucidating the precise mechanisms of GV‐971 in the treatment of NMOSD and evaluating its therapeutic potential in clinical settings.

## AUTHOR CONTRIBUTIONS

Z.X., S.C., and M.G. conceived of and supervised the research. M.G., S.C., Z.X., and J.Z. provided the resources. Z.X., X.Y., Z.Z., G.S., and J.Z. designed the methodology. X.Y., Z.Z., A.Y., Q.Z., A.X., J.Q., M.C., X.C., L.L, Z.F., Z.L., and G.S. performed the investigation. X.Y., Z.Z., X.C., and Z.L. wrote the manuscript. Z.X. and S.C. edited the manuscript.

## CONFLICT OF INTEREST STATEMENT

X.Y. A.X., J.Q., M.C., X.C., L.L, Z.F., Z.L., G.S., and J.Z. are employees of Shanghai Green Valley Pharmaceutical Co., Ltd.

## CONSENT FOR PUBLICATION

Not applicable.

## Supporting information


Figures S1–S2

Tables S1–S3

## Data Availability

The data that support the findings of this study are available on request from the corresponding author. The data are not publicly available due to privacy or ethical restrictions.

## References

[1] Wingerchuk DM , Lennon VA , Lucchinetti CF , Pittock SJ , Weinshenker BG . The spectrum of neuromyelitis optica. Lancet Neurol. 2007;6(9):805‐815. 17706564 10.1016/S1474-4422(07)70216-8

[2] Fiala C , Rotstein D , Pasic MD . Pathobiology, diagnosis, and current biomarkers in Neuromyelitis Optica Spectrum disorders. J Appl Lab Med. 2022;7(1):305‐310. 34996075 10.1093/jalm/jfab150

[3] Wingerchuk DM , Banwell B , Bennett JL , et al. International consensus diagnostic criteria for neuromyelitis optica spectrum disorders. Neurology. 2015;85(2):177‐189. 26092914 10.1212/WNL.0000000000001729 PMC4515040

[4] Du Q , Shi Z , Chen H , et al. Mortality of neuromyelitis optica spectrum disorders in a Chinese population. Ann Clin Transl Neurol. 2021;8(7):1471‐1479. 34120408 10.1002/acn3.51404 PMC8283162

[5] Akaishi T , Takahashi T , Misu T , et al. Progressive patterns of neurological disability in multiple sclerosis and neuromyelitis optica spectrum disorders. Sci Rep. 2020;10(1):13890. 32807848 10.1038/s41598-020-70919-w PMC7431838

[6] Ma X , Kermode AG , Hu X , Qiu W . NMOSD acute attack: understanding, treatment and innovative treatment prospect. J Neuroimmunol. 2020;348:577387. 32987231 10.1016/j.jneuroim.2020.577387

[7] Lennon VA , Kryzer TJ , Pittock SJ , Verkman AS , Hinson SR . IgG marker of optic‐spinal multiple sclerosis binds to the aquaporin‐4 water channel. J Exp Med. 2005;202(4):473‐477. 16087714 10.1084/jem.20050304 PMC2212860

[8] Weinshenker BG , Wingerchuk DM , Pittock SJ , Lucchinetti CF , Lennon VA . NMO‐IgG: a specific biomarker for neuromyelitis optica. Dis Markers. 2006;22(4):197‐206. 17124341 10.1155/2006/586306 PMC3851409

[9] Wang HH , Dai YQ , Qiu W , et al. Interleukin‐17‐secreting T cells in neuromyelitis optica and multiple sclerosis during relapse. J Clin Neurosci. 2011;18(10):1313‐1317. 21795048 10.1016/j.jocn.2011.01.031

[10] Varrin‐Doyer M , Spencer CM , Schulze‐Topphoff U , et al. Aquaporin 4‐specific T cells in neuromyelitis optica exhibit a Th17 bias and recognize clostridium ABC transporter. Ann Neurol. 2012;72(1):53‐64. 22807325 10.1002/ana.23651 PMC3405197

[11] Barros PO , Cassano T , Hygino J , et al. Prediction of disease severity in neuromyelitis optica by the levels of interleukin (IL)‐6 produced during remission phase. Clin Exp Immunol. 2016;183(3):480‐489. 26472479 10.1111/cei.12733 PMC4750605

[12] Agasing AM , Wu Q , Khatri B , et al. Transcriptomics and proteomics reveal a cooperation between interferon and T‐helper 17 cells in neuromyelitis optica. Nat Commun. 2020;11(1):2856. 32503977 10.1038/s41467-020-16625-7 PMC7275086

[13] Matsuya N , Komori M , Nomura K , et al. Increased T‐cell immunity against aquaporin‐4 and proteolipid protein in neuromyelitis optica. Int Immunol. 2011;23(9):565‐573. 21795759 10.1093/intimm/dxr056

[14] Liu J , Zhang Q , Shi Z , et al. Increased expression of the membrane‐bound CD40 ligand on peripheral CD4(+) T cells in the acute phase of AQP4‐IgG‐seropositive neuromyelitis optica spectrum disorders. J Neuroimmunol. 2018;325:64‐68. 30408708 10.1016/j.jneuroim.2018.10.013

[15] Chang H , Cong H , Wang H , et al. Thymic involution and altered naive CD4 T cell homeostasis in Neuromyelitis Optica Spectrum disorder. Front Immunol. 2021;12:645277. 34335563 10.3389/fimmu.2021.645277 PMC8322781

[16] Thomas DL , Manners J , Marker D , et al. CD8‐positive T‐cell Leukoencephalitis with Astrocytopathy clinically presenting as Neuromyelitis Optica. J Neuropathol Exp Neurol. 2017;76(5):347‐357. 28340257 10.1093/jnen/nlx015

[17] Shi Z , Qiu Y , Zhao Z , et al. CD8(+) T cell subpopulations and pro‐inflammatory cytokines in neuromyelitis optica spectrum disorder. Ann Clin Transl Neurol. 2021;8(1):43‐53. 33231379 10.1002/acn3.51241 PMC7818084

[18] Fan X , Jiang Y , Han J , et al. Circulating memory T follicular helper cells in patients with Neuromyelitis Optica/Neuromyelitis Optica Spectrum disorders. Mediat Inflamm. 2016;2016:3678152. 10.1155/2016/3678152 PMC4804098 27057097

[19] Yang X , Peng J , Huang X , et al. Association of circulating follicular helper T cells and serum CXCL13 with Neuromyelitis Optica Spectrum disorders. Front Immunol. 2021;12:677190. 34335576 10.3389/fimmu.2021.677190 PMC8316915

[20] Brill L , Lavon I , Vaknin‐Dembinsky A . Foxp3+ regulatory T cells expression in neuromyelitis optica spectrum disorders. Mult Scler Relat Disord. 2019;30:114‐118. 30771576 10.1016/j.msard.2019.01.047

[21] Cree BA , Spencer CM , Varrin‐Doyer M , Baranzini SE , Zamvil SS . Gut microbiome analysis in neuromyelitis optica reveals overabundance of Clostridium perfringens. Ann Neurol. 2016;80(3):443‐447. 27398819 10.1002/ana.24718 PMC5053302

[22] Gong J , Qiu W , Zeng Q , et al. Lack of short‐chain fatty acids and overgrowth of opportunistic pathogens define dysbiosis of neuromyelitis optica spectrum disorders: a Chinese pilot study. Mult Scler. 2019;25(9):1316‐1325. 30113252 10.1177/1352458518790396

[23] Cui C , Tan S , Tao L , et al. Intestinal barrier breakdown and mucosal microbiota disturbance in Neuromyelitis optical Spectrum disorders. Front Immunol. 2020;11:2101. 32983166 10.3389/fimmu.2020.02101 PMC7492665

[24] Shi Z , Qiu Y , Wang J , et al. Dysbiosis of gut microbiota in patients with neuromyelitis optica spectrum disorders: a cross sectional study. J Neuroimmunol. 2020;339:577126. 31841737 10.1016/j.jneuroim.2019.577126

[25] Pandit L , Cox LM , Malli C , et al. Clostridium bolteae is elevated in neuromyelitis optica spectrum disorder in India and shares sequence similarity with AQP4. Neurol Neuroimmunol Neuroinflamm. 2021;8(1):e907. 33148687 10.1212/NXI.0000000000000907 PMC7643530

[26] Zhang J , Xu YF , Wu L , Li HF , Wu ZY . Characteristic of gut microbiota in southeastern Chinese patients with neuromyelitis optica spectrum disorders. Mult Scler Relat Disord. 2020;44:102217. 32534438 10.1016/j.msard.2020.102217

[27] Hoyles L , Pontifex MG , Rodriguez‐Ramiro I , et al. Regulation of blood‐brain barrier integrity by microbiome‐associated methylamines and cognition by trimethylamine N‐oxide. Microbiome. 2021;9(1):235. 34836554 10.1186/s40168-021-01181-z PMC8626999

[28] Braniste V , Al‐Asmakh M , Kowal C , et al. The gut microbiota influences blood‐brain barrier permeability in mice. Sci Transl Med. 2014;6(263):263ra158. 10.1126/scitranslmed.3009759 PMC4396848 25411471

[29] Papadopoulos MC , Verkman AS . Aquaporin 4 and neuromyelitis optica. Lancet Neurol. 2012;11(6):535‐544. 22608667 10.1016/S1474-4422(12)70133-3 PMC3678971

[30] Chen T , Noto D , Hoshino Y , Mizuno M , Miyake S . Butyrate suppresses demyelination and enhances remyelination. J Neuroinflammation. 2019;16(1):165. 31399117 10.1186/s12974-019-1552-y PMC6688239

[31] Jing Y , Yu Y , Bai F , et al. Effect of fecal microbiota transplantation on neurological restoration in a spinal cord injury mouse model: involvement of brain‐gut axis. Microbiome. 2021;9(1):59. 33678185 10.1186/s40168-021-01007-y PMC7937282

[32] Xiao S , Chan P , Wang T , et al. A 36‐week multicenter, randomized, double‐blind, placebo‐controlled, parallel‐group, phase 3 clinical trial of sodium oligomannate for mild‐to‐moderate Alzheimer's dementia. Alzheimers Res Ther. 2021;13(1):62. 33731209 10.1186/s13195-021-00795-7 PMC7967962

[33] Wang T , Kuang W , Chen W , et al. A phase II randomized trial of sodium oligomannate in Alzheimer's dementia. Alzheimers Res Ther. 2020;12(1):110. 32928279 10.1186/s13195-020-00678-3 PMC7489025

[34] Syed YY . Sodium Oligomannate: first approval. Drugs. 2020;80(4):441‐444. 32020555 10.1007/s40265-020-01268-1

[35] Wang X , Sun G , Feng T , et al. Sodium oligomannate therapeutically remodels gut microbiota and suppresses gut bacterial amino acids‐shaped neuroinflammation to inhibit Alzheimer's disease progression. Cell Res. 2019;29(10):787‐803. 31488882 10.1038/s41422-019-0216-x PMC6796854

[36] Lu J , Pan Q , Zhou J , et al. Pharmacokinetics, distribution, and excretion of sodium oligomannate, a recently approved anti‐Alzheimer's disease drug in China. J Pharm Anal. 2022;12:145‐155. 35573885 10.1016/j.jpha.2021.06.001 PMC9073255

[37] Zhou C , Zhang J , Luo X , et al. Sodium Oligomannate electrostatically binds to Abeta and blocks its aggregation. J Phys Chem B. 2023;127(9):1983‐1994. 36848623 10.1021/acs.jpcb.3c00280

[38] Kong LN , Geng MY , Mu L , Xin XL , Yang N , Zuo PP . Effects of acidic oligose on differentially expressed genes in the mice model of Alzheimer's disease by microarray. Yao Xue Xue Bao. 2005;40(12):1105‐1109. 16496674

[39] Fan Y , Hu J , Li J , et al. Effect of acidic oligosaccharide sugar chain on scopolamine‐induced memory impairment in rats and its related mechanisms. Neurosci Lett. 2005;374(3):222‐226. 15663967 10.1016/j.neulet.2004.10.063

[40] Hu J , Geng M , Li J , et al. Acidic oligosaccharide sugar chain, a marine‐derived acidic oligosaccharide, inhibits the cytotoxicity and aggregation of amyloid beta protein. J Pharmacol Sci. 2004;95(2):248‐255. 15215650 10.1254/jphs.fpj04004x

[41] Jiang RW , Du XG , Zhang X , et al. Synthesis and bioassay of beta‐(1,4)‐D‐mannans as potential agents against Alzheimer's disease. Acta Pharmacol Sin. 2013;34(12):1585‐1591. 24241344 10.1038/aps.2013.104 PMC4002563

[42] Yu Z , Yang Y , Chan RB , et al. GV‐971 attenuates alpha‐Synuclein aggregation and related pathology. CNS Neurosci Ther. 2023;30:e14393. 37563872 10.1111/cns.14393 PMC10848097

[43] Xiang W , Xie C , Luo J , et al. Low frequency ultrasound with injection of NMO‐IgG and complement produces lesions different from experimental autoimmune encephalomyelitis mice. Front Immunol. 2021;12:727750. 34721390 10.3389/fimmu.2021.727750 PMC8551829

[44] Saadoun S , Waters P , Bell BA , Vincent A , Verkman AS , Papadopoulos MC . Intra‐cerebral injection of neuromyelitis optica immunoglobulin G and human complement produces neuromyelitis optica lesions in mice. Brain. 2010;133(Pt 2):349‐361. 20047900 10.1093/brain/awp309 PMC2822632

[45] Gao J , Zhao X , Hu S , et al. Gut microbial DL‐endopeptidase alleviates Crohn's disease via the NOD2 pathway. Cell Host Microbe. 2022;30(10):1435‐1449 e1439. 36049483 10.1016/j.chom.2022.08.002

[46] Nakai M , Ribeiro RV , Stevens BR , et al. Essential hypertension is associated with changes in gut microbial metabolic pathways: a multisite analysis of ambulatory blood pressure. Hypertension. 2021;78(3):804‐815. 34333988 10.1161/HYPERTENSIONAHA.121.17288

[47] Ewels PA , Peltzer A , Fillinger S , et al. The nf‐core framework for community‐curated bioinformatics pipelines. Nat Biotechnol. 2020;38(3):276‐278. 32055031 10.1038/s41587-020-0439-x

[48] Martin M . Cutadapt removes adapter sequences from high‐throughput sequencing reads. EMBnet J. 2011;17(1):10‐12.

[49] Fang P , Kazmi SA , Jameson KG , Hsiao EY . The microbiome as a modifier of neurodegenerative disease risk. Cell Host Microbe. 2020;28(2):201‐222. 32791113 10.1016/j.chom.2020.06.008 PMC7430034

[50] Cerovic M , Forloni G , Balducci C . Neuroinflammation and the gut microbiota: possible alternative therapeutic targets to counteract Alzheimer's disease? Front Aging Neurosci. 2019;11:284. 31680937 10.3389/fnagi.2019.00284 PMC6813195

[51] Yarandi SS , Peterson DA , Treisman GJ , Moran TH , Pasricha PJ . Modulatory effects of gut microbiota on the central nervous system: how gut could play a role in neuropsychiatric health and diseases. J Neurogastroenterol Motil. 2016;22(2):201‐212. 27032544 10.5056/jnm15146 PMC4819858

[52] Foster JA , McVey Neufeld KA . Gut‐brain axis: how the microbiome influences anxiety and depression. Trends Neurosci. 2013;36(5):305‐312. 23384445 10.1016/j.tins.2013.01.005

[53] Ding H , Wang G , Yu Z , Sun H , Wang L . Role of interferon‐gamma (IFN‐gamma) and IFN‐gamma receptor 1/2 (IFNgammaR1/2) in regulation of immunity, infection, and cancer development: IFN‐gamma‐dependent or independent pathway. Biomed Pharmacother. 2022;155:113683. 36095965 10.1016/j.biopha.2022.113683

[54] Mehmood A , Shah S , Guo RY , et al. Methyl‐CpG‐binding protein 2 emerges as a central player in multiple sclerosis and Neuromyelitis Optica Spectrum disorders. Cell Mol Neurobiol. 2023;43(8):4071‐4101. 37955798 10.1007/s10571-023-01432-7 PMC11407427

[55] Matsushita T , Tateishi T , Isobe N , et al. Characteristic cerebrospinal fluid cytokine/chemokine profiles in neuromyelitis optica, relapsing remitting or primary progressive multiple sclerosis. PLoS One. 2013;8(4):e61835. 23637915 10.1371/journal.pone.0061835 PMC3630114

[56] Khan Z , Mehan S , Gupta GD , Narula AS . Immune system dysregulation in the progression of multiple sclerosis: molecular insights and therapeutic implications. Neuroscience. 2024;548:9‐26. 38692349 10.1016/j.neuroscience.2024.04.004

[57] Hou MM , Li YF , He LL , et al. Proportions of Th17 cells and Th17‐related cytokines in neuromyelitis optica spectrum disorders patients: a meta‐analysis. Int Immunopharmacol. 2019;75:105793. 31401379 10.1016/j.intimp.2019.105793

[58] Lin J , Li X , Xia J . Th17 cells in neuromyelitis optica spectrum disorder: a review. Int J Neurosci. 2016;126(12):1051‐1060. 10.3109/00207454.2016.1163550 26954363

[59] Wang K , Song F , Fernandez‐Escobar A , Luo G , Wang JH , Sun Y . The properties of cytokines in multiple sclerosis: pros and cons. Am J Med Sci. 2018;356(6):552‐560. 30447707 10.1016/j.amjms.2018.08.018

[60] Hiltensperger M , Korn T . The interleukin (IL)‐23/T helper (Th)17 Axis in experimental autoimmune encephalomyelitis and multiple sclerosis. Cold Spring Harb Perspect Med. 2018;8(1):a029637. 29101111 10.1101/cshperspect.a029637 PMC5749145

[61] Andreadou M , Ingelfinger F , De Feo D , et al. IL‐12 sensing in neurons induces neuroprotective CNS tissue adaptation and attenuates neuroinflammation in mice. Nat Neurosci. 2023;26(10):1701‐1712. 37749256 10.1038/s41593-023-01435-z PMC10545539

[62] Ting AT , Bertrand MJM . More to life than NF‐kappaB in TNFR1 signaling. Trends Immunol. 2016;37(8):535‐545. 27424290 10.1016/j.it.2016.06.002 PMC5076853

[63] Carney DE , Lutz CJ , Picone AL , et al. Soluble tumor necrosis factor receptor prevents post‐pump syndrome. J Surg Res. 1999;83(2):113‐121. 10329104 10.1006/jsre.1999.5576

[64] Idriss HT , Naismith JH . TNF alpha and the TNF receptor superfamily: structure‐function relationship(s). Microsc Res Tech. 2000;50(3):184‐195. 10891884 10.1002/1097-0029(20000801)50:3<184::AID-JEMT2>3.0.CO;2-H

[65] Lee H , Park S , Ju S , et al. Preparation and evaluation of colon‐targeted prodrugs of the microbial metabolite 3‐Indolepropionic acid as an Anticolitic agent. Mol Pharm. 2021;18(4):1730‐1741. 33661643 10.1021/acs.molpharmaceut.0c01228

[66] Owumi SE , Adebisi G . Epirubicin treatment induces neurobehavioral, Oxido‐inflammatory and Neurohistology alterations in rats: protective effect of the endogenous metabolite of tryptophan – 3‐Indolepropionic acid. Neurochem Res. 2023;48(9):2767‐2783. 37097396 10.1007/s11064-023-03941-9

[67] Amarakoon D , Lee WJ , Tamia G , Lee SH . Indole‐3‐Carbinol: occurrence, health‐beneficial properties, and cellular/molecular mechanisms. Annu Rev Food Sci Technol. 2023;14:347‐366. 36972159 10.1146/annurev-food-060721-025531

[68] Jeelan Basha N , Basavarajaiah SM , Shyamsunder K . Therapeutic potential of pyrrole and pyrrolidine analogs: an update. Mol Divers. 2022;26(5):2915‐2937. 35079946 10.1007/s11030-022-10387-8 PMC8788913

[69] Fitzpatrick PF . The enzymes of microbial nicotine metabolism. Beilstein J Org Chem. 2018;14:2295‐2307. 30202483 10.3762/bjoc.14.204 PMC6122326

[70] Schadlich IS , Winzer R , Stabernack J , Tolosa E , Magnus T , Rissiek B . The role of the ATP‐adenosine axis in ischemic stroke. Semin Immunopathol. 2023;45(3):347‐365. 36917241 10.1007/s00281-023-00987-3 PMC10279578

