# Supplementary material for: GV‐971 attenuates the progression of neuromyelitis optica in murine models and reverses alterations in gut microbiota and associated peripheral abnormalities
Source: CNS Neurosci Ther. 2024 Jul 7;30(7):e14847. doi: 10.1111/cns.14847 (PMC11228355; doi:10.1111/cns.14847)
Supplement: Supplementary file 1 — Figures S1–S2 [file CNS-30-e14847-s001.docx]

**
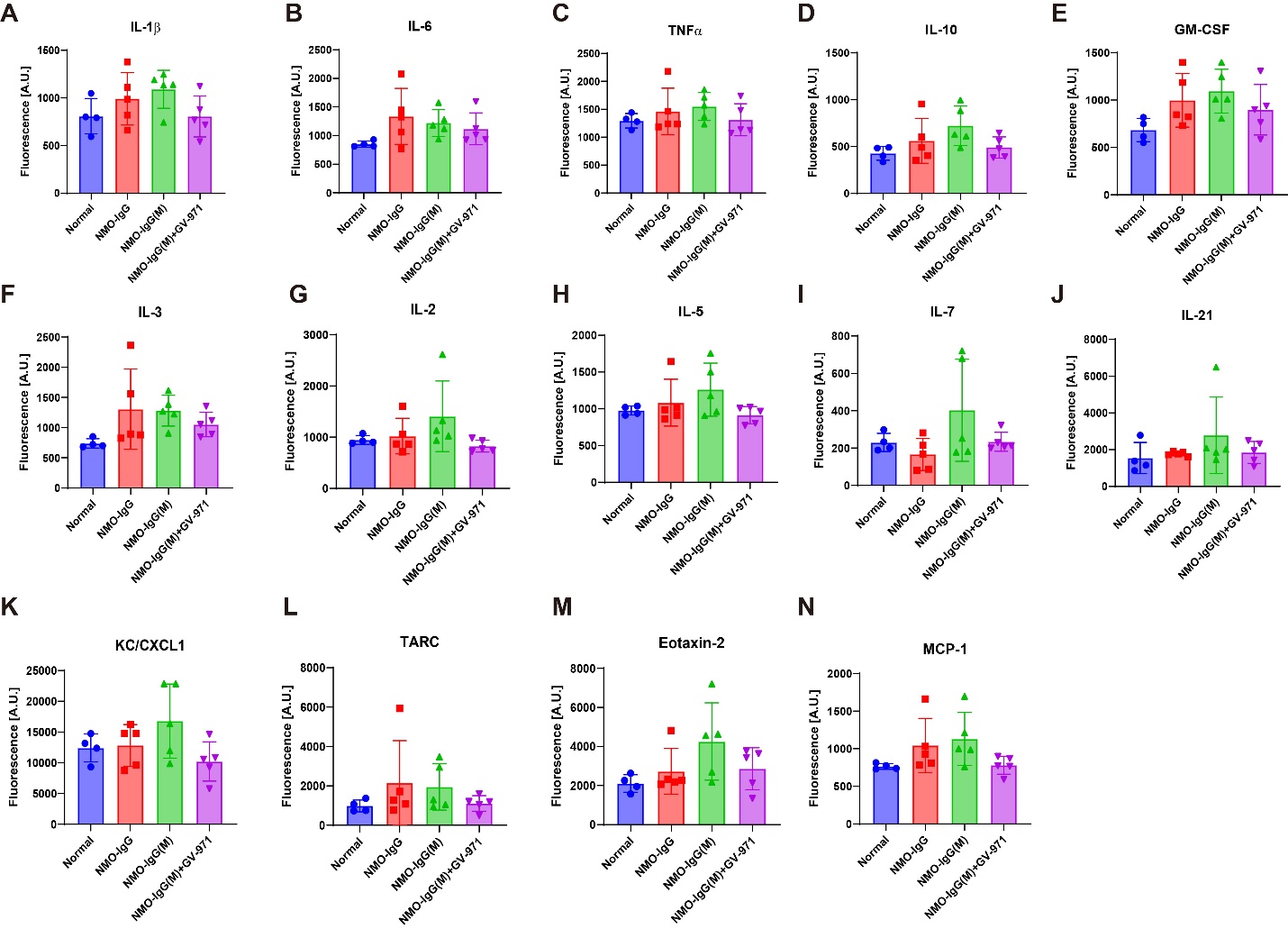
Figure S1.** Effect of GV-971 on plasma cytokines in NMOSD model mice. (A-N) Fluorescence intensity (a.u.) of plasma cytokines in each group. Data presented as Mean±S.E.M. One-way *ANOVA*, ^*^*P*<0.05.

**
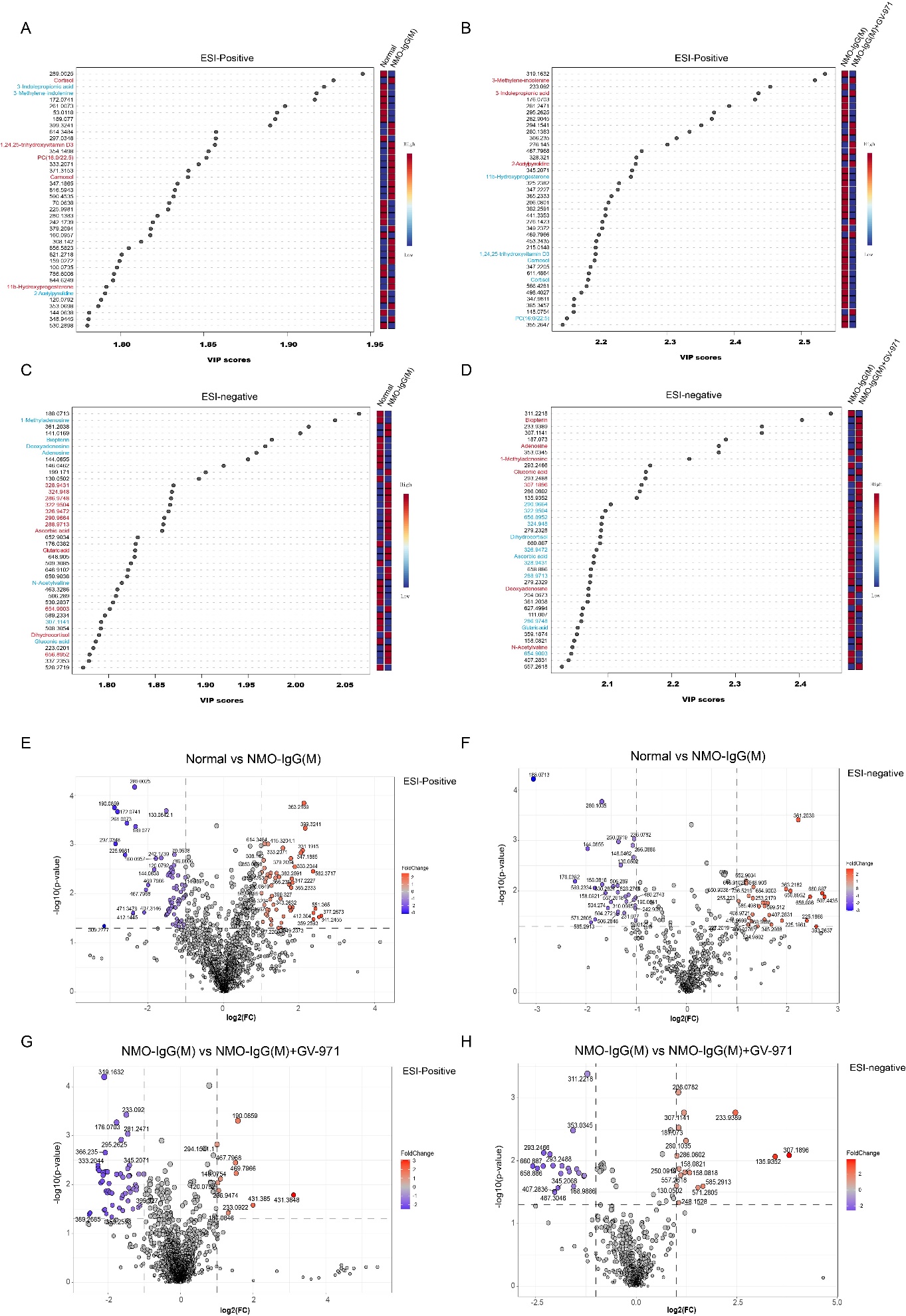
**

**Figure S2.** The differential metabolites between different groups. (A) The top40 discriminative metabolites between NMO-IgG(M) and normal groups in ESI-positive mode using PLS-DA analysis. (B) The top40 discriminative metabolites between NMO-IgG(M)+GV-971 and NMO-IgG(M) groups in ESI-positive mode using PLS-DA analysis. (C) The top40 discriminative metabolites between NMO-IgG(M) and normal groups in ESI-negative mode using PLS-DA analysis. (D) The top40 discriminative metabolites between NMO-IgG(M)+GV-971 and NMO-IgG(M) groups in ESI-negative mode using PLS-DA analysis. (E) Volcano plot significantly changed metabolites between NMO-IgG(M) and normal groups in ESI-positive mode. (F) Volcano plot significantly changed metabolites between NMO-IgG(M) and normal groups in ESI-negative mode. (G) Volcano plot significantly changed metabolites between NMO-IgG(M)+GV-971 and NMO-IgG(M) groups in ESI-positive mode. (H) Volcano plot significantly changed metabolites between NMO-IgG(M)+GV-971 and NMO-IgG(M) groups in ESI-negative mode.
